# Cushing Syndrome due to a Pancreatic Neuroendocrine Tumor Treated With Radiofrequency Ablation

**DOI:** 10.1210/jcemcr/luad022

**Published:** 2023-03-09

**Authors:** Aristea Sideri Gugger, Jiali Fang, Kavel H Visrodia, Gabrielle Page-Wilson

**Affiliations:** Department of Medicine, Columbia University Vagelos College of Physicians & Surgeons, Columbia University Irving Medical Center, New York, NY 10032, USA; Department of Medicine, Columbia University Vagelos College of Physicians & Surgeons, Columbia University Irving Medical Center, New York, NY 10032, USA; Department of Medicine, Columbia University Vagelos College of Physicians & Surgeons, Columbia University Irving Medical Center, New York, NY 10032, USA; Department of Medicine, Columbia University Vagelos College of Physicians & Surgeons, Columbia University Irving Medical Center, New York, NY 10032, USA

**Keywords:** Cushing syndrome, ectopic ACTH syndrome, endocrine neoplasia, neuroendocrine tumor, proopiomelanocortin, radiofrequency ablation

## Abstract

Delayed diagnosis of Cushing syndrome (CS) results in advanced disease, treatment delays, and poor outcomes. We present a patient with ectopic ACTH syndrome (EAS) from a pancreatic neuroendocrine tumor (NET) whose care posed diagnostic and therapeutic challenges. A 59-year-old female with classic Cushing stigmata, biochemical evidence of ACTH-dependent hypercortisolism, and a 5-mm pituitary lesion presented for inferior petrosal sinus sampling, which was contraindicated due to non-ST elevation myocardial infarction and acute/subacute strokes. Whole-body computed tomography (CT) scan was unrevealing, but elevations in chromogranin A and proopiomelanocortin (POMC) concentrations suggested EAS. Positron emission tomography-CT with gallium 68-DOTATATE demonstrated a 7-mm pancreatic tail lesion, suspicious for a pancreatic NET. The patient was not a surgical candidate and treatment with ketoconazole was complicated by hepatoxicity. Endoscopic ultrasound-guided biopsy and radiofrequency ablation of the lesion was pursued. Pathology confirmed ACTH immunoreactive low-grade pancreatic NET. Post procedure, sustained normalization of ACTH and cortisol was achieved. This case supports the utility of POMC measurements in the differential diagnosis of CS and the use of advanced nuclear imaging for tumor localization. For patients with functional pancreatic NET who are poor surgical candidates or intolerant of pharmacotherapy, novel endoscopic ablation may offer a low-risk therapeutic option and should be further investigated.

## Introduction

Endogenous Cushing syndrome (CS) is a rare, debilitating disorder characterized by chronic hypercortisolism and metabolic, cardiovascular, musculoskeletal, and psychiatric signs and symptoms. Diagnosis is often delayed due to symptomatic overlap with other common metabolic disorders, contributing to disease progression, treatment delays, and poor outcomes [1]. The majority of CS cases are adrenocorticotropic hormone (ACTH)-dependent, arising from corticotroph pituitary adenomas (Cushing disease), and less frequently from ectopic neuroendocrine tumors including thymic, pancreatic, and bronchial carcinoids; pheochromocytomas; and medullary thyroid and small cell lung carcinomas [1]. Differentiating between Cushing disease (CD) and ectopic ACTH syndrome (EAS) can be challenging, given that the culprit tumors are often small and difficult to locate, and the clinical and biochemical presentations of EAS and CD may be similar.

Pituitary magnetic resonance imaging (MRI) is the first diagnostic step following confirmation of ACTH-dependent CS. Patients with pituitary adenomas ≥ 10 mm are presumed to have CD. For those with adenomas < 6 to 10 mm, bilateral inferior petrosal sinus sampling (IPSS), which involves sampling blood for ACTH from the pituitary venous runoff at the base of the brain, is the gold standard test for distinguishing between CD and EAS [2]. However, access to IPSS is limited and contraindications exist [2]. In the absence of IPSS, neuroendocrine peptide measurements [3, 4] and advanced nuclear imaging studies [2] can be considered in diagnostic dilemmas. Once the tumor is localized, surgical resection is recommended. Pharmacologic therapies, including steroidogenesis inhibitors, tumor directed medications, and glucocorticoid receptor antagonists can be used for patients who are not surgical candidates, but they are not curative treatment options and may be associated with side effects [2].

Here, we present a diagnostically and therapeutically challenging case of a patient with EAS from a pancreatic neuroendocrine tumor (NET) who ultimately achieved biochemical remission with a novel minimally invasive procedure—endoscopic ultrasound-guided (EUS) radiofrequency ablation (RFA).

## Case Presentation

**Figure 1.**
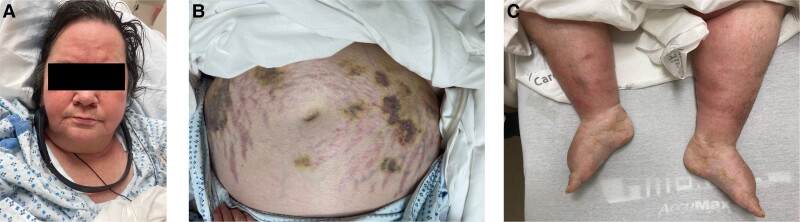
Patient appearance on admission. A. Facial plethora, flushing, and hair loss, B. Abdominal obesity with large purple striae and overlying ecchymoses, C. Lower extremity edema and additional ecchymoses.

A 59-year-old woman was transferred for evaluation and management of ACTH-dependent CS. The patient had a 2-year history of an unexplained 50-pound weight gain, facial plethora, hair thinning, easy bruising, and severe proximal muscle weakness limiting ambulation, paired with new onset hypertension and type 2 diabetes mellitus on multiple medications. Past medical history included obesity, hyperlipidemia, remote 30 pack-year smoking history, nonalcoholic fatty liver disease, nephrolithiasis, severe osteoporosis with multiple vertebral compression fractures, and a recent non-ST elevation myocardial infarction (NSTEMI). Physical examination was notable for a Cushingoid appearing woman with a body mass index of 48 kg/m^2^, moon facies, facial plethora, supraclavicular and dorsocervical fat pads, obese abdomen with large purple striae, lower extremity edema, limited movement of extremities against gravity and numerous ecchymoses (Fig. 1).

## Diagnostic Assessment

**Figure 2.**
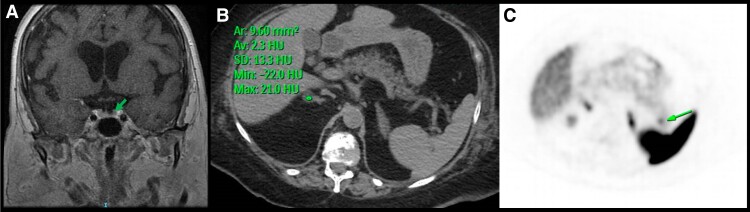
A, Pituitary MRI: Possible 0.5-cm lesion and acute/subacute CVAs. B, Chest/abdominal/pelvis CT: 1.5-cm lipid-rich adrenal adenoma. C, ^68^Ga-DOTATATE PET-CT scan: pancreatic tail 7-mm DOTATATE-avid lesion, suspicious for NET.

Laboratory assessment demonstrated ACTH-dependent CS (Table 1). Pituitary MRI (3T) revealed a possible 0.5-cm anterior pituitary lesion (Fig. 2A) and acute/subacute strokes, which precluded IPSS. Computed tomography (CT) of chest/abdomen/pelvis was notable for a 1.5-cm R lipid-rich adrenal adenoma (2.3 Hounsfield units) (Fig. 2B). The radiographic appearance excluded adrenocortical carcinoma, and biochemical workup ruled out pheochromocytoma and hyperaldosteronism. Since the adrenal nodule was not felt to be the culprit lesion, further workup was pursued. Serum chromogranin A (CGA), and plasma proopiomelanocortin (POMC) [3], the ACTH precursor, were elevated, favoring the diagnosis of EAS (Table 1). A subsequent positron emission tomography (PET)-CT scan with gallium 68-DOTATATE revealed a 7-mm DOTATATE-avid (SUV_max_ 14.5) lesion in the pancreatic tail, suspicious for a somatostatin receptor (SSTR)-expressing pancreatic NET (Fig. 2C).

**Table 1.** Pre-admission evaluation and additional biochemical workup obtained during hospitalization for ACTH-dependent CS

Diagnostic testing	Results	Normal range
Pre-admission workup of ACTH-dependent Cushing syndrome		
Baseline	ACTH: 96 pg/mL	7.2-63.3 pg/mL
	Serum cortisol: 19.6 mcg/dL	4.8-19.5 mcg/dL
1 mg overnight dexamethasone suppression test (ODST)	Serum cortisol: 18.5 mcg/dL	<1.8 mcg/dL
	Dexamethasone: 547 ng/dL	40-295 ng/dL
24-hour urine free cortisol (UFC)	Urine cortisol total: 192	<45 mcg/dL
	Urine creatinine total: 484 mg/day	
Late-night salivary cortisol (LNSC)	Sample 1: 436 Sample 2: 464	29-101 ng/dL
8 mg high-dose dexamethasone suppression tests (HDDST)	Serum cortisol: 7.2 mcg/dL*^a^*	<1.8 mcg/dL
Additional biochemical workup obtained during hospitalization		
ACTH	82.5 pg/mL	7.2-63.3 pg/mL
Serum cortisol	18.5 mcg/dL	4.8-19.5 mcg/dL
POMC	154 fmol/mL	< 40 fmol/mL
Serum cortisol	18.5 mcg/dL	4.8-19.5 mcg/dL
Chromogranin A	538 ng/mL	0-103 ng/mL
Pancreatic polypeptide	798 pg/mL	0-435 pg/mL
24 hour urine 5-hydroxyindoleacetic acid (5HIAA)/Cr	3 mg/24h	0-15 mg/24h

1 mg ODST, 24-hour UFC, and LNSC tests all demonstrate hypercortisolism.

a HDDST with greater than 50% suppression of cortisol levels.

## Treatment

**Figure 3.**
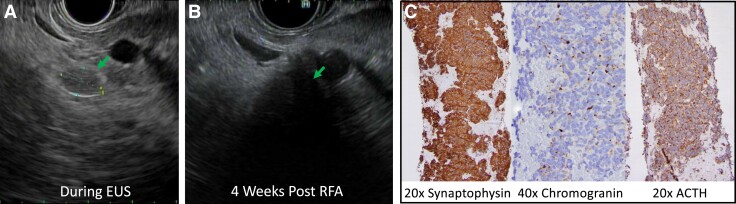
A, EUS image of tumor pre-RFA. B, Same area EUS image 4 weeks post RFA shows replacement of tumor with necrosis. C, Tumor stained for synaptophysin, chromogranin A, and ACTH.

Partial pancreatectomy was recommended, but the patient was not a surgical candidate. Medical therapy with ketoconazole was initiated and an excellent biochemical response was achieved, but due to drug-induced hepatotoxicity (Table 2), ketoconazole was discontinued. The off-label use of osilodrostat was planned, but given challenges obtaining the medication, a multidisciplinary meeting was held to discuss other therapeutic options, and the decision was made to pursue EUS-guided fine needle aspiration followed by RFA of the pancreatic lesion. The procedure was performed under general anesthesia but without endotracheal intubation. A 9-mm lesion was endosonographically identified in the pancreatic tail (Fig. 3A) and core tissue was sampled using a 22-gauge fine needle biopsy. A dedicated 19-gauge radiofrequency ablation catheter (EUSRA RFA Electrode STARmed, Koyang, Korea) was then used to puncture the lesion after ensuring major vessels and the pancreatic duct were not in proximity to the lesion. Three total RFA treatments were delivered in one session, each at 10 Watts, resulting in minimal residual appearing lesion. The procedure was well tolerated, without complications. Biopsy confirmed ACTH immunoreactive low-grade pancreatic NET (Fig. 3C).

**Table 2.** 24-hour urine free cortisol and liver enzymes prior to and during treatment with ketoconazole

	Before treatment with ketoconazole	During treatment with ketoconazole
24-hour urine free cortisol (<45 mcg/d)	192	23
Total bilirubin (0.2-1.3 mg/dL)	0.4	0.3
Direct bilirubin (0.0-0.3 mg/dL)	0.2	0.1
AST (10-37 U/L)	19	247
ALT (9-50 U/L)	25	160
Alkaline phosphatase (40-129 ug/L)	191	739
Gamma-glutamyl transferase (9-58 U/L)	280	819

Abbreviations: ALT, alanine transaminase; AST, aspartate aminotransferase.

## Outcome and Follow-up

Following the procedure, morning ACTH and cortisol were 8 pg/mL and 9.1 mcg/dL respectively, and glucocorticoids were required to treat relative adrenal insufficiency. Day 28 post procedure, a repeat EUS showed necrotic appearing changes replacing the previously ablated lesion (Fig 3B). RFA was not repeated.

The patient was discharged to a rehabilitation facility. Two months later, a repeat biochemical evaluation showed remission of CS (Table 3). Ten months post procedure, the patient remained in remission and demonstrated remarkable clinical improvement including a 100-pound weight loss and resolution of diabetes (HbA1c 5.5% off all antidiabetic medications, down from 7.7%). Her hypertension was well controlled with a single agent.

**Table 3.** Pre- and post-RFA neuroendocrine laboratory values supporting biochemical remission

	ACTH (7.2-63.3 pg/mL)	Serum cortisol (4.8-19.5 mcg/dL)	UFC excretion (<45 ug/d)	Cr excretion (ug/24 hours)	LNSC (<100 ng/dL)	POMC (<40 fmol/mL)	Chromogranin A (0-103 ng/mL)
Pre-RFA	74.1	27.56	115	470	464	154	538
2 months post-RFA	28.3	17.8	12	354	50	23	72
10 months postRFA		6.2			<50		

Abbreviations: ACTH, adrenocorticotropic hormone; Cr, creatinine; LNSC, late-night salivary cortisol, POMC, proopiomelanocortin; RFA, radiofrequency ablation; UFC, urinary free cortisol.

## Discussion

The differential diagnosis of ACTH-dependent CS remains one of the more challenging issues in clinical endocrinology and contraindications to gold standard diagnostic and treatment modalities may further complicate the evaluation and management. This case highlights the effective use of novel, minimally invasive, diagnostic and therapeutic tools. The first step in identifying the source of ACTH-secretion is tumor localization. Traditional biochemical tests, including corticotropin releasing hormone and desmopressin stimulation tests, can be useful for distinguishing between pituitary and ectopic tumors, but corticotropin releasing hormone is costly and not widely available, and both tests have diagnostic limitations. Although the high-dose dexamethasone suppression test is easy to perform, it lacks sensitivity and specificity [5], as illustrated by this case. Our patient exhibited >50% suppression of serum cortisol in response to an 8-mg dexamethasone load, which classically indicates the presence of CD; however, she was ultimately diagnosed with EAS. Standard imaging modalities also have limitations; only 30% to 50% of the pituitary tumors that cause CD are detected by MRI [2]. Furthermore, given that incidental pituitary microadenomas affect 10% of the population [6], the presence of a microadenoma is not sufficient for the diagnosis of CD, as it may be a red herring as in this case. While IPSS is the gold standard test for distinguishing between pituitary and ectopic ACTH-secreting tumors, this invasive and technically rigorous procedure is not widely available, false-positive/negative results have been described, and complications including subarachnoid hemorrhage and brain stem injury can occur in rare cases [7]. Furthermore, as demonstrated by this case, contraindications to IPSS exist, including bleeding disorders, recent stroke, ischemic heart disease, contrast allergy, orthopnea, or inability to lay flat [7]. Given these limitations, other diagnostic tools that capitalize on tumor-specific differences in peptide processing and synthesis may prove clinically useful.

In occult Cushing, elevations in plasma POMC, the precursor to ACTH, have been shown to aid in the differential diagnosis, with POMC levels >36 fmol/mL exhibiting a sensitivity and specificity for EAS of 64% and 100%, respectively [3]. The accumulation of POMC precursors in EAS results from the fact that poorly differentiated ectopic tumors exhibit aberrant POMC processing, while well-differentiated ACTH-secreting pituitary microadenomas process the peptide precursor more efficiently, resulting in relatively low circulating POMC concentrations [3]. While the POMC assay is not currently available in commercial clinical laboratories, it can be accessed through research laboratories [3]. CGA is secreted by neuroendocrine cells and elevations can indicate the presence of NETs [4]. This nonspecific biomarker can also add value in the differential diagnosis of Cushing, but it must be interpreted with caution as elevations can be caused by proton pump inhibitors, renal impairment, hypertension, and other conditions [4]. In our case, since IPSS was contraindicated, and thin-slice whole-body CT did not reveal a culprit tumor, elevations in POMC and CGA aided the diagnostic evaluation, supporting the likelihood of EAS and the use of advanced imaging modalities. ^68^Ga-DOTATATE binds to somatostatin receptors and when used with PET-CT localizes approximately 65% of the NETs that cause EAS, including those not detected by CT or MRI, and it is more sensitive for detecting smaller tumors than traditional octreotide scans [2].

First-line treatment for EAS is tumor resection. Unfortunately, our patient was not a surgical candidate due to complications of long-standing, untreated Cushing. Medical therapy is a second-line treatment alternative and can serve as a bridge to eventual curative interventions. However, the medications used to treat Cushing can have drug interactions and side effects and in our patient's case, hepatotoxicity prompted medication discontinuation. EUS RFA is an emerging modality for treatment of various pancreatic lesions, including pancreatic NETs [8]. EUS RFA involves the use of a through-the-scope needle to puncture the lesion and deliver high-frequency alternating current to induce focal hyperthermia (temperatures 60-100 °C) and necrosis of the lesion with minimal injury to surrounding tissue. Its use in liver tumors is well-established and based on increasing data supporting its favorable safety and efficacy profile in small (< 2 cm) pancreatic NETs, along with its minimally invasive approach, EUS-guided RFA offers an attractive alternative for patients requiring treatment for pancreatic NETs who are not surgical candidates [8].

We identified 2 case reports of Cushing syndrome treated with EUS-guided RFA. In the first case, EAS due to a 7-mm ACTH-secreting endobronchial carcinoid was treated with RFA as a bridge to lobectomy [9]. In the second case, a non-ACTH-dependent 2.8-cm cortisol-producing adrenal nodule required repeated attempts at RFA prior to temporary biochemical remission [10]. To our knowledge, this is the first case describing the use of RFA to treat ectopic Cushing syndrome from a pancreatic neuroendocrine tumor; however, our gastroenterology colleagues had previous experience using EUS-guided RFA to treat other pancreatic neuroendocrine tumors, including insulinomas [8]. In this case, EUS-guided RFA was a minimally invasive procedure that the patient could safely tolerate, and it successfully led to a biochemical remission.

In conclusion, this case highlights the application of novel diagnostics and therapeutics for the management of ACTH-dependent Cushing syndrome. POMC is a noninvasive serum test that can help differentiate between CD and EAS in cases where IPSS is unavailable or contraindicated. In patients with functional NET who are not surgical candidates or who are intolerant of pharmacotherapy, endoscopic RFA may offer a low-risk therapeutic alternative and its clinical use deserves further investigation.

## Learning Points

- Incidental pituitary microadenomas affect 10% of the population, and 30% to 50% of patients with Cushing disease (CD) have no discernible pituitary lesions on MRI, complicating differential diagnosis of ACTH-dependent Cushing syndrome (CS).
- Inferior petrosal sinus sampling (IPSS) is the gold standard test for differentiating CD from ectopic ACTH syndrome, but it is available only at select centers and for suitable candidates. When IPSS is unavailable or contraindicated, neuroendocrine markers (chromogranin A and POMC) can aid in the differential diagnosis, and ^68^Ga-DOTATATE PET-CT can facilitate tumor localization.
- For patients with functional pancreatic NET who are not surgical candidates or exhibit medication intolerance, EUS-guided RFA may be a low-risk minimally invasive therapeutic alternative.

## Data Availability

Data sharing is not applicable to this article as no datasets were generated or analyzed during the current study.

## Abbreviations

ACTH: adrenocorticotropic hormone
CD: Cushing disease
CGA: chromogranin A
CS: Cushing syndrome
EAS: ectopic ACTH syndrome
EUS: endoscopic ultrasound
IPSS: inferior petrosal sinus sampling
MRI: magnetic resonance imaging
NET: neuroendocrine tumor
POMC: proopiomelanocortin
RFA: radiofrequency ablation
